# No Significant Bone Resorption after Open Treatment of Mandibular Condylar Head Fractures in the Medium-Term

**DOI:** 10.3390/jcm11102868

**Published:** 2022-05-19

**Authors:** Michael-Tobias Neuhaus, Nils-Claudius Gellrich, Anna Katharina Sander, Bernd Lethaus, Dirk Halama, Rüdiger M. Zimmerer

**Affiliations:** 1 Department of Oral and Maxillofacial Surgery, Leipzig University, Liebigstraße 12, 04103 Leipzig, Germany; anna.sander@medizin.uni-leipzig.de (A.K.S.); bernd.lethaus@medizin.uni-leipzig.de (B.L.); dirk.halama@medizin.uni-leipzig.de (D.H.); ruediger.zimmerer@medizin.uni-leipzig.de (R.M.Z.); 2 Department of Oral and Maxillofacial Surgery, Hannover Medical School, Carl-Neuberg-Str. 1, 30625 Hannover, Germany; gellrich.nils-claudius@mh-hannover.de

**Keywords:** condylar head fractures, intraarticular fractures, ORIF, open treatment, bone remodeling, mandibular condyle fractures, CHF

## Abstract

Open treatment of condylar head fractures (CHF) is considered controversial. In this retrospective cohort study our primary objective was therefore to assess bone resorption and remodeling as well as patients function after open treatment of CHF in a medium-term follow-up (15.1 ± 2.2 months). We included 18 patients with 25 CHF who underwent open reduction and internal fixation, between 2016 and 2021, in our analysis. The clinical data and cone-beam computed tomography (CBCT) datasets were analyzed. The condylar processes were segmented in the postoperative (T1) and follow-up (T2) CBCT scans. Volumetric and linear bone changes were the primary outcome variables, measured by using a sophisticated 3D-algorithm. The mean condylar head volume decreased non-significantly from 3022.01 ± 825.77 mm^3^ (T1) to 2878.8 ± 735.60 mm^3^ (T2; *p* = 0.52). Morphological alterations indicated remodeling and resorption. The pre-operative maximal interincisal opening (MIO) was 19.75 ± 3.07 mm and significantly improved to 40.47 ± 1.7 mm during follow-up (*p* = 0.0005). Low rates of postoperative complications were observed. Open reduction of CHF leads to good clinical outcomes and low rates of medium-term complications. This study underlines the feasibility and importance of open treatment of CHF and may help to spread its acceptance as the preferred treatment option.

## 1. Introduction

Mandibular fractures are the most common craniofacial fractures with 25–45% of cases involving the condylar process [1,2]. The treatment of these condylar fractures has been controversial for years. For displaced fractures located at the condylar neck and base, open treatment has become “gold standard” [3,4,5] with superior function and outcomes compared to closed treatment; however, for condylar head fractures the discourse is ongoing. Despite existing nomenclature systems [2,6,7], there is often no distinct differentiation between the types of condylar process fractures made in the literature [8,9,10] which has resulted in Nussbaum et al. concluding that a meta-analysis is not feasible due to the inconsistency of reported fractures and nomenclature in the underlying studies [11]. This is made more convoluted due to the different levels of fracture of the condylar process representing completely different, non-comparable types of diseases, morbidity, complications, and outcomes. The latest IBRA position paper did not clearly recommend open treatment for condylar head fractures (CHF) [3]; however, in more recent studies, open treatment has been suggested and seems to be superior to closed treatment [3,12,13,14,15]. In a randomized cohort study of closed vs. open treatment, the surgically treated patients with condylar head fractures had superior function, pain, and occlusion [16] In the authors’ opinion, the so-called closed reduction or conservative treatment is misleading because a closed repositioning of the fractured and displaced condylar head against muscular traction seems highly unlikely to occur. Furthermore, necessary immobilization of the fractured TMJ is troublesome in the aspect of ankylosis or reduced mouth opening. Open treatment of displaced CHF is considered the only way to achieve long-term restoration of function and anatomically stable results with reconstruction of vertical height, adequate joint function and prevention of occlusal disturbances [14].

Osteosynthesis with two positional screws has proven to be appropriate for treating CHF [17]. Although the removal of osteosynthesis material was formerly only recommended in cases of implant failure [18,19], some of the latest studies using volumetric and 3D evaluation of bone resorption strongly recommend standardized removal 4 to 6 months after surgery to prevent screw protrusion and reduce the rate of subsequent joint disorders [14,15,20,21].

Previous studies investigated bone resorption after open treatment of CHF using different techniques and made adverse conclusions in terms of screw/plate removal [15,19,20]. The resorption seems to affect the lateral and dorsal aspects of the condylar head, rather than vertical height and articulating surface [20,22,23].

The purpose of this study was to evaluate bone remodeling and resorption in a patient cohort after open treatment of CHF. The authors hypothesize, that open treatment of CHF, despite bone remodeling, would lead to good clinical outcomes and improve patients’ function. The specific aims were: (1) 3D volumetric analysis of bone remodeling, (2) analysis of clinical outcome, and (3) verification of necessity of routinely osteosynthesis removal.

## 2. Materials and Methods

### 2.1. Study Design

In this retrospective two-center analysis, the Department of Oral and Maxillofacial Surgery, Hannover Medical School, Hannover, Germany, and the Department for Oral and Maxillofacial Surgery, Leipzig University Hospital, Leipzig, Germany, were screened for patients who presented with CHF between 2016 and 2020. Patients had to meet the following inclusion criteria:

1. Displaced condylar head fracture type M or P according to Neff et al. [2], regardless of the degree of fragmentation (none, minor, major) and vertical apposition (complete, partial, or lost).
2. Open reduction and internal fixation (ORIF) were performed using positional screws with or without additional mini-plate osteosynthesis.
3. Complete patient documentation during follow-up of at least 6 months.
4. Postoperative and follow-up cone beam computed tomography (CBCT) scans of appropriate quality allowing for 3D-segmentation.
5. Fulfilled patient consent. Patients were excluded if they were lost to a follow-up.

This study was approved by the local ethics review committee (Hannover Medical School; study no.: 8163_BO_K_2018). The analyses were performed in accordance with the declaration of Helsinki. All included patients provided informed consent.

### 2.2. Variables

#### 2.2.1. Clinical Parameters and Outcome Measures

In this study the primary outcome variable was the postoperative bone remodeling of the condylar head. Secondary outcome variables (covariables) were: TMJ-related parameters, such as maximal interincisal opening (MIO); laterotrusion; deviation; occlusion; TMJ-related pain; and joint noises (T1: intra- or immediately postoperatively; T2: follow-up examinations). Covariates were: age, sex, and surgical parameters, such as duration of operation, fracture comminution and displacement, and osteosynthesis type.

#### 2.2.2. Surgical Procedure

A preauricular approach was used to access the CHF. After incision of skin and subcutaneous fatty tissue, the temporalis fascia was identified and incised in a 60° angle towards the zygomatic arch. Beneath the fascia, the zygomatic arch was dissected subperiosteally while the plane of dissection was continued on the articular capsule. In order to enter the lower joint space, the articular capsule was incisied in a T-like manner. The upper joint space was left untouched, as was the disk. The lateral aspect of the condylar head and the condylar neck were exposed, just enough to allow for adequate open reduction and internal fixation. Reduction was performed under anesthesiological relaxation and the usually medio-caudally displaced fractured condylar head was reduced using specialized instruments including condylar hooks and pliers. Standard osteosynthesis used in this study were two 1.5 mm positioning screws of about 16–18 mm in length. In cases of major fragmentation more screws or additional plates were used and in cases of additional mandible fractures, the CHF was treated first. During wound closure the TMJ capsule was closed with resorbable suture, as well as the temporalis fascia. In the majority of cases, no MMF was installed. Postoperatively, patients were treated with early functional physiotherapy.

### 2.3. Data Collection Methods

#### 2.3.1. 3D Segmentation and Analysis

CBCT scans were performed in clinical routine intra- or immediately postoperatively (T1) for verification of repositioning and positioning of osteosynthesis, as well as during the follow-up examinations (T2).

**Figure 1:**
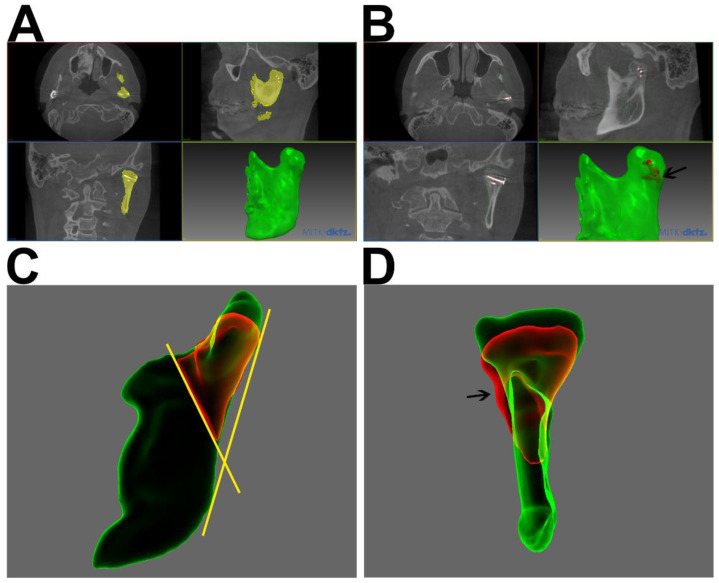
(**A**) Follow-up CBCT-scan of a 21-year-old male patient with a bilateral fracture of the condylar head. Segmentation of the left mandibular ramus in MITK Workbench ^®^ (yellow) and the generated smoothed STL-file (green) are shown. (**B**) Same patient as (**A**) with segmented and visualized osteosynthesis material (black arrow, three positioning screws). (**C**) Lateral view on the superimposition of segmentations of CBCT-scans at T1 (green) and T2 (red) (Artec Studio^®^); condylar neck of T2 (red) already split at the defined line (yellow) between most inferior point of the sigmoid notch and the most posterior boundary of the mandibular foramen. (**D**) Dorsal view of (**C**); remodeling of the condylar head is clearly visible with loss of height. Especially at the lateral aspect callus and resorption at the screw head are visible (black arrow).

**Figure 2:**
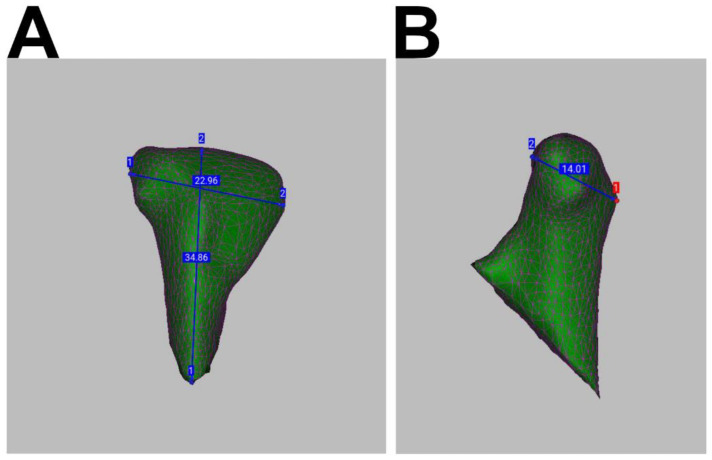
(**A**) Dorsal view on a follow up (T2) CBCT-scan segmentation of the left condylar neck of a 32-year-old male patient. Linear measurements at the greatest extent in transverse and longitudinal orientation are shown, performed in Artec Studio^®^. (**B**) Lateral view on the same segmentation as in (**A**) with linear measurement in the sagittal orientation.

For the 3D analysis of condylar heads, the whole mandibular rami were segmented, using MITK Workbench (German Cancer Research Center [DKFZ] Division of Medical Image Computing, Heidelberg, Germany). DICOM data sets were imported into MITK Workbench, which features an “Otsu” algorithm for automated threshold-based segmentation [24]. A standardized configuration was applied (nine regions and 70 histogram bins). Segmentations were corrected and processed using morphological operations such as gap closing and hole filling. Smoothed STL files were generated and exported (Figure 1A,B). To reduce osteosynthesis-related scattering, osteosynthesis material was segmented, visualized and, if necessary, subtracted (Figure 1B). Volumetric and metric measurements were processed in Artec^®^ Studio 15 (Luxembourg, Luxembourg). To separate the condylar processes, the 3D-segmentations of T1 and T2 were imported, superimposed, and cut at a line through the most inferior point of the sigmoid notch and the posterior boundary of the mandibular foramen (Figure 1C,D). Setting a cut line closer to the fractured, and potentially irregular, condylar head might be prone to error; however, the superimposition of the postoperative (T1) and follow-up (T2) segmentations prior to cutting allowed for splits at exactly the same line. This further reduces any possible methodological volume deviations. The volumes of the isolated condylar processes were calculated using Artec^®^ Studio 15. For metric measurements of remodeling, a Cartesian coordinate system was projected onto the condylar head to determine the offset between T1 and T2 in the transverse, sagittal, and longitudinal orientations (Figure 2).

#### 2.3.2. Data Analysis

Statistical analyses were performed using Microsoft Excel^®^ 2019 (Microsoft, Redmond, WA, USA) and ‘R’ (The R Foundation for Statistical Computing, c/o Institute for Statistics and Mathematics, Wirtschaftsuniversität Wien, Vienna, Austria). Means, standard deviations, and medians were calculated, and t-tests were used to compare values. *p* values < 0.05 were considered statistically significant.

## 3. Results

Screening of department databases yielded 30 patients meeting the inclusion criteria, of which 12 patients were lost to follow-up. Eighteen patients with eligible datasets at both time points were included in this study. These patients led to a total of 25 openly treated CHF. The mean follow-up period was 15.1 ± 2.2 month with a median of 14-months.

### 3.1. Demographics and Etiology

Sex distribution was nearly even with 11 male (61.1%) and 7 female (38.9%) patients. Their ages at the date of surgery ranged from 17 to 76 years, with a mean of 43.1 ± 3.9 years. Falls were the most common cause of trauma, followed by traffic accidents

### 3.2. Fracture Topography and Classification

All fractures were condylar head fractures according to AO classification [2]. In total, 7 fractures (38.9%) were bilateral, and 10 were associated with additional mandibular fractures (55.6%) (Table 1). Comminuted fractures with more than two condylar head fragments occurred in 61.1% of the fractures.

### 3.3. Clinical and Surgical Parameters

Preoperative maximal interincisal opening (MIO) was reduced to 19.75 ± 3.07 mm. It significantly improved to 40.47 ± 1.7 mm during follow up (*p* = 0.0005) (Table 1). Patient’s pain improved as well from 5.0 ± 0.61 (VAS) preoperative to 0.33 ± 0.19 during follow up (*p* = 0.008). Deviation during mouth opening was observed in five patients during follow-up. These five cases were solely unilateral fractures, with intraarticular screws in follow-up CBCT in three cases. In one case a secondary material removal was performed, this was also recommended in another two cases, but was rejected by the patients. Another case with jaw deviation underwent primary surgery 4 weeks after trauma, due to severe occlusal disturbance. The mean laterotrusion of both sides was 5.56 ± 0.83 mm. No patient showed severe TMJ dysfunction, and only one patient showed an occlusal disturbance. No further long-term complications, such as facial nerve palsy, salivary fistulae, or clinically apparent arthrosis, occurred.

The mean surgical time was 120.09 ± 9.03 min per fracture. Most commonly for fracture osteosynthesis, two positioning screws were used (60.0%), followed by three positioning screws (12%), and one 4-hole osteosynthesis plate (12%) (Table 1). Seven patients showed joint noise during the follow-up (38.33%). Standard osteosynthesis materials with screw diameters between 1.5 and 2.0 mm were used.

#### Morphological Alterations of the Condylar Head

**Figure 3:**
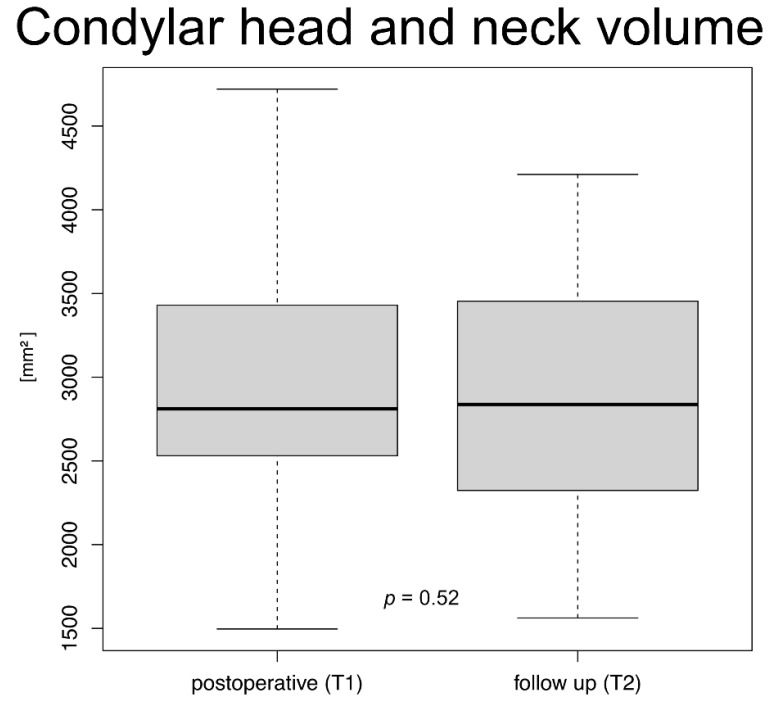
Boxplot of condylar neck volume [mm^3^] postoperative (T1) and follow up (T2); no significant difference between both examinations was seen, *p* = 0.52 (median, box height: interquartile range, whiskers: lowest and highest value).

Mean condylar head volume decreased from 3022.01 ± 825.77 mm^3^ at T1 to 2878.8 ± 735.60 mm^3^ at T2, however this difference was not significant (*p* = 0.52) (Figure 3). The mean volume difference was 143.21 ± 465.72 mm^3^. Mean mandibular ramus height changed by 1.55 ± 1.65 mm (*p* = 0.172). Mean transversal condyle width changed by 1.162 ± 1.96 mm (*p* = 0.086) and mean sagittal condyle width changed by 0.45 ± 1.601 mm^3^ (*p* = 0.575). All changes in the linear measurements were not statistically significant. Morphological alterations in CBCT-scans at T2, however, commonly indicated remodeling and resorption (Table 2). Three patients underwent secondary surgery and plate removal after the follow-up CBCT (T2).

## 4. Discussion

The purpose of this study was to assess bone remodeling and resorption after open treatment of CHF. The hypothesis was that the open treatment leads to good clinical outcome and patients’ function.

**Figure 4:**
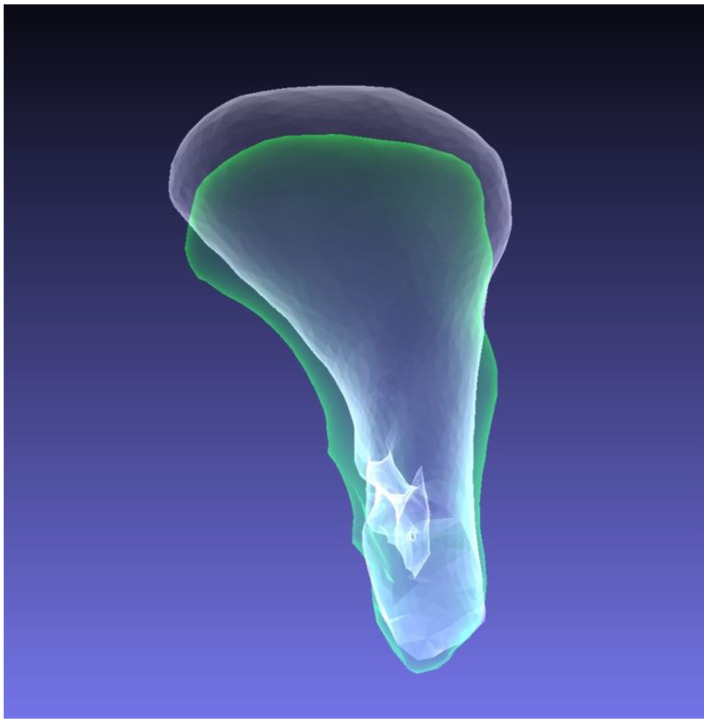
Dorsal view on translucent illustration of fusion of preoperative (white) and follow-up (green) CBCT segmentations. Fusion has been performed with complete mandibular segmentation. For illustration reasons, the mandibular condyle has been separated at a formerly defined line as presented in Figure 1C.

In CBCT scans during follow-up, remodeling of the fractured condylar heads was frequently observed (Table 1), even though there was no significant change in the condylar head volumes and linear dimensions. These findings match those of previous studies on this topic, which could not prove any significant volumetric changes either [15,20]. Johner et al. observed a volumetric decrease of 15.29%, without any significance as well [15]. In our study the mandibular ramus height decreased by a median of 0.84 mm without being statistically significant; however, a similar amount of loss of vertical ramus height was observed in previous studies [19,20,22]. The qualitative assessment of 3D datasets showed resorption in 14 cases (Table 1). Apparently, in those cases the condylar head seemed to be lower, and in some cases, wider (in transverse orientation) (Figure 1D and Figure 4). Unfortunately, those qualitative findings could not be objectified as mentioned before. Further, qualitative alterations in the condylar head configuration, such as arthrosis or callus and sequester did not have any correlation with clinical symptoms or TMJ-function.

The following analyses are unlikely due to the small sample size: influence of comminution, dislocation, fracture location, and the presence of additional fractures on resorption. The studies mentioned below were also unable to demonstrate statistically significant correlations [14,20].

Contrary to other authors, we do not recommend a general removal of osteosynthesis material. For this reason, it was not performed in our study. Only in cases with osteosynthesis failure or intraarticular screws was it recommended to the patient. This procedure is supported by Smolka et al. [19]. However, the removal of osteosynthesis remains the point of discussion. Therefore, the need of secondary surgery (screw/plate removal) in 16% of cases, considering the low overall risk for complications and risk for facial nerve palsy, seems justifiable. The future use of resorbable osteosynthesis materials could potentially avoid these secondary procedures [25,26,27].

There are several previous studies on bone remodeling after surgical treatment of CHF [15,19,20], each of which uses a different analytical approach. Recent studies have described either 3D or volumetric measurements; therefore, we decided to combine both measurements of volumetric alterations and 2D changes of the condylar head, which were modified according to Skroch et al. 2020 [20]. By using sophisticated automated segmentation algorithms in the present study, the volume bias during segmentation is reduced. The splitting of mandibular segmentations for volumetric assessment has been modified in this study, as we have seen a bias in the determination of the condylar head baseline due to fracture-related alterations in anatomy. The previously described projection of a sphere into the pole zone of the condylar head could be prone to bias [2]; therefore, we set the cutting line lower, through the most inferior point of the sigmoid notch and the posterior boundary of the mandibular foramen (Figure 1) to prevent interference with osteosynthesis material and trauma-related deformities of the condylar head and neck. These points are reliably recognizable during the segmentation process. This does not affect the total volumetric resorption measurements, but the calculated relative resorption could be estimated to be lower as the reference volume increased. During segmentation and volume measurement, osteosynthesis material outside the condylar heads was excluded. As no surgery to the TJR occurred between T1 and T2 in this study, another reason for bias could be avoided.

Most other studies compared patients’ TMJ function using the well-established Helkimo index. This index was initially described in 1978 by Helkimo [28] for the classification of patients with temporomandibular joint disorders. Since then, it has been widely used for the classification and documentation of any kind of TMJ disorder, including fractures of the TMJ; however, this index does not sufficiently reflect the characteristics of patients with TMJ fracture or deformities in the course of TMJ trauma. To mitigate this, we used MIO, laterotrusion, and joint pain separately to compare patients’ functions.

A latest clinical trial showed that open treatment seems to be clearly superior to conservative treatment in terms of clinical function [29]. The information on clinical function and outcome in our study is limited due to its medium-term follow-up of 15 months. This challenge could be clearly observed throughout the literature as the rate of patients lost to follow-up increases over time, with common follow-up periods of 5 to 6 months [19,20,29]. Xie et al. [18] were able to observe their patients over a period of 24 months. They could clearly show a stagnation in improvement of TMJ-function as well as bone resorption after 12 to 24 months. This, in our study, allowed for implications on remodeling and function, even after a median follow-up of 15 month.

Our clinical findings show significant improvements in TMJ function and pain between T1 and T2, with no severe TMJ disorders or medium-term complications at T2. Despite the anesthesiologic risk, additional expenses, and necessary surgeon training, our study findings support the open treatment of CHF and the findings of previous studies with low resorption and good clinical patient outcomes [14,16,19,20,29]. Therefore, we strongly recommend the open treatment for displaced condylar head fractures, as it is still not widely performed.

## 5. Conclusions

In conclusion, open treatment of condylar head fractures leads to low bone resorption with no significant alterations in the course after surgery. Patients show good TMJ-function and outcomes with low complication rates. These findings, as well as the latest literature, promote the open reduction and osteosynthesis of condylar head fractures. However, further prospective clinical trials need to be conducted and the shortcomings of a retrospective approach need to be overcome, before this trend can be ascertained.

## Figures and Tables

**Table 1:** Fracture classification.

Clinical Parameters	No. of Cases	[%]
	Total n = 18	
Cause of trauma		
Fall	12	66.7
Traffic accident	5	27.8
Violence	1	5.6
Fracture classification		
Non/slightly displaced	1	5.6
Displaced	13	72.2
Comminuted and displaced	11	61.1
Additional fractures		
Median	7	38.9
Paramedian	3	16.7
Collum	3	16.7
Total cases with additional fractures	10	55.6
Osteosynthesis type		
2 positioning screws	15	60.0
3 positioning screws	3	12.0
1 × 4-hole plate	3	12.0
2 plates	2	8.0
Others	2	8.0
Follow up CBCT diagnosis		
No change	6	24.0
Resorption	14	56.0
Arthrosis	3	12.0
Osteosynthesis failure	1	4.0
Intraarticular screw	4	16.0
Sequester	1	4.0
Callus	4	16.0

CBCT: Cone beam computed tomography.

**Table 2:** Patients’ function and surgery time.

Clinical Parameters	Mean	SEM	*p*
MIO preop [mm]	19.71	3.51	
MIO follow up [mm]	40.47	1.70	0.0001
Laterotrusion follow up [mm]	5.56	0.83	
Pain preop (VAS)	5.00	0.61	
Pain follow up (VAS)	0.33	0.19	0.008
Surgical time pre fracture [min]	120.09	9.03	

MIO: maximal interincisal opening.

## Data Availability

Not applicable.
